# Targeting *Salmonella* Typhimurium Invasion and Intracellular Survival Using Pyrogallol

**DOI:** 10.3389/fmicb.2021.631426

**Published:** 2021-02-02

**Authors:** Biruk Tesfaye Birhanu, Eon-Bee Lee, Seung-Jin Lee, Seung-Chun Park

**Affiliations:** ^1^Laboratory of Veterinary Pharmacokinetics and Pharmacodynamics, College of Veterinary Medicine, Kyungpook National University, Daegu, South Korea; ^2^Development and Reproductive Toxicology Research Group, Korea Institute of Toxicology, Daejeon, South Korea

**Keywords:** intracellular inhibition, invasion, marbofloxacin, pharmacodynamic, pyrogallol

## Abstract

*Salmonella enterica* serovar Typhimurium, an intracellular pathogen, evades the host immune response mechanisms to cause gastroenteritis in animals and humans. After invading the host cells, the bacteria proliferate in *Salmonella*-containing vacuole (SCV) and escapes from antimicrobial therapy. Moreover, *Salmonella* Typhimurium develops resistance to various antimicrobials including, fluoroquinolones. Treating intracellular bacteria and combating drug resistance is essential to limit the infection rate. One way of overcoming these challenges is through combination therapy. In this study, Pyrogallol (PG), a polyphenol, is combined with marbofloxacin (MAR) to investigate its effect on *Salmonella* Typhimurium invasion and intracellular survival inhibition. The Minimum inhibitory concentration (MIC) and minimum bactericidal concentration (MBC) of PG against *Salmonella* Typhimurium were 128 and 256 μg/mL, respectively. The lowest fractional inhibitory concentration (FIC) index for a combination of PG and MAR was 0.5. The gentamycin protection assay revealed that PG (30 μg/mL) alone and in combination with sub-MIC of MAR inhibited 72.75 and 76.18% of the invading bacteria in Caco-2 cells, respectively. Besides, the intracellular survival of *Salmonella* Typhimurium was reduced by 7.69 and 74.36% in treatment with PG alone and combined with sub-MIC of MAR, respectively, which was visualized by the confocal microscopy. PG has also shown to increase the intracellular accumulation of fluoroquinolone by 15.2 and 34.9% at 30 and 100 μg/mL concentration, respectively. Quantitative real-time PCR demonstrated PG suppressed the genetic expression of *hilA, invF, sipB*, and *acrA* by 14.6, 15.4, 13.6, and 36%, respectively. However, the downregulation of *hilA, invF, sipB*, and *acrA* increased to 80, 74.6, 78, and 70.1%, in combination with sub-MIC of MAR, respectively. Similarly, PG combined with MAR inhibited the expression of *sdiA, srgE*, and *rck* genes by 78.6, 62.8, and 61.8%, respectively. In conclusion, PG has shown antimicrobial activity against *Salmonella* Typhimurium alone and in combination with MAR. It also inhibited invasion and intracellular survival of the bacteria through downregulation of quorum sensing, invading virulence, and efflux pump genes. Hence, PG could be a potential antimicrobial candidate which could limit the intracellular survival and replication of *Salmonella* Typhimurium.

## Introduction

*Salmonella enterica* subsp. *enterica* serovar Typhimurium, an intracellular Gram-negative bacterium, is a non-typhoidal Salmonella serotype known to cause diarrhea and intestinal inflammation in humans and animals (Laupland et al., 2010). Upon ingestion, salmonella adhere to epithelial lining of the ilium and colon to cause gastrointestinal infection (Ribet and Cossart, 2015). In the small intestine, the bacteria invade specialized epithelial cells called M cells. It triggers membrane ruffling and actin rearrangement which leads to bacterial internalization upon injecting its effector proteins (Patel and Galán, 2005). The T3SS-1 genes plays a significant role to invade the intestinal epithelial cells and mediate bacterial entry and their persistence in the host cells (Thiennimitr et al., 2012). Successful adherence and invasion of the host cell are essential to cause infections. *Salmonella* Typhimurium invades epithelial cells either through cytoskeletal rearrangement known as the “Trigger” mechanism or receptor-mediated entry “Zipper” mechanism (Velge et al., 2012). Internalization is facilitated by several *Salmonella* secreted effector proteins (Agbor and Mccormick, 2011). After internalization *Salmonella* resides in the *Salmonella*-containing vacuoles to shield from host innate immune responses (Haraga et al., 2008). Hence, inhibition of bacterial adhesion, invasion, and intracellular survival is critical in controlling infections. However, effective delivery of antimicrobial agents within the host cells is the main challenge (Kamaruzzaman et al., 2017). Hence, identifying natural compounds with a higher penetration capacity has significant importance (Liu et al., 2020).

Besides, *Salmonella* Typhimurium developed resistance against fluoroquinolones, including marbofloxacin (MAR), which is a widely used antibacterial agent against intracellular bacteria in veterinary medicine (de Jong et al., 2012). Antimicrobial resistance has become a common trend worldwide, and most of the bacteria have been developing resistance to the commonly utilized antibacterial agents (Varma et al., 2005). To worsen the situation, the introduction of newly invented antimicrobials into the market is declining. Hence, designing alternative methods to overcome these challenges is critical. One means of tackling the alarmingly overgrowing drug resistance is through a combination therapy of currently available antimicrobials, either with other existing antimicrobials or using natural compounds (Fischbach, 2011).

Polyphenols are phytochemicals found richly in natural, edible plants. They are known for their antioxidant properties, antibacterial activity, and prevention of non-communicable diseases like cancer, cardiovascular, and neurodegenerative diseases (Manach et al., 2004; Taguri et al., 2006). Furthermore, polyphenols inhibit angiogenesis, and aging, maintains blood pressure and sugar level, and have anti-inflammatory properties, regulate enzyme function, and stimulate cell receptors (Middleton et al., 2000; Pandey and Rizvi, 2009; Moyle et al., 2015). Recently, polyphenols like methyl gallate showed to prevent microbial adhesion, invasion, and intracellular survival of microbial agents through inhibition of biofilm formation and quorum sensing signals (Hossain et al., 2017; Birhanu et al., 2018).

Pyrogallol (benzene-1,2,3-triol, PG) is a hydroxylated polyphenol compound that contains three hydroxyl groups in the ortho position of a benzene ring. It is also present in various fruits and vegetables. PG is mostly used in pharmaceutical and pesticide manufacturing companies for medicinal purposes as a topical antipsoriatic (Ozturk Sarikaya, 2015). PG exhibits both prooxidant and antioxidant activity. The earlier role of PG involves generating reactive oxygen species and critical for its antimicrobial activity (Baruah et al., 2015; Mendes et al., 2015). Besides, PG acts through enzymatic inhibition of oxidized compounds (Mason and Bruce, 1987).

Previously PG showed to inhibit *Helicobacter pylori* urease (Xiao et al., 2010) and *Vibrio harveyi* quorum sensing (Ni et al., 2008). Its acethlycholinesterase inhibitory activity shows its importance in treating Alzheimer’s disease (Ozturk Sarikaya, 2015). Moreover, PG prevents the HeLa cells cytotoxicity induced by *V. vulnificus* and inhibits bacterial growth (Lim et al., 2016). Furthermore, it showed a synergistic activity when combined with norfloxacin and gentamicin against *Staphylococcus aureus* (Lima et al., 2016).

However, to our understanding, there are no reports on the effect of PG on bacterial intracellular survival and its antiinvasive mechanism. Hence, in this study, we evaluated the pharmacodynamics of PG and its inhibitory activity against *Salmonella* Typhimurium invasion and intracellular survival alone and in combination with MAR. Moreover, we have studied the mechanisms of bacterial invasion and intracellular survival inhibition.

## Materials and Methods

### Bacterial and Cell Culture Conditions

Two field strains of *Salmonella enterica* serovar Typhimurium, (KU325552 (S-2) and KU325565 (S-15), isolated from swine clinical infections as described earlier (Lee et al., 2017), in which one is susceptible while the other was intermediately resistant to MAR (Fluka, Germany) and ATCC 14028 strain were used. All the bacteria were cultured in Luria-Bertani (LB) broth or agar (Difco, BD, MD, United States) unless otherwise stated, at 37^*o*^C for various time lengths according to the purpose of the experiment. Two cell lines, RAW 264.7 and Caco-2 cells were used for the bacterial invasion and intracellular survival assays. RAW 264.7 cells were grown in RPMI 1640 medium (Sigma, United Kingdom) supplemented with 10% fetal bovine serum (FBS, Gibco, United States) and 1% penicillin-streptomycin (P/S) whereas, the Caco-2 cell was grown in minimum essential medium (MEM, Gibco, United States) supplemented with 1% nonessential amino acids (Sigma-Aldrich, MO, United States), 20% FBS, and 1% P/S and both cell lines were incubated at 37°C in a humidified atmosphere with 5% CO_2_.

### Cell Viability Assay

The cell viability assay was performed using 3-(4,5-dimethyl-2-thiazolyl)-2,5-diphenyl-2*H*-tetrazolium bromide (MTT) assay. Confluent cells (10^5^ cells/mL) were cultured onto a 96-well plate and incubated for 24 h at 37^*o*^C with 5% CO_2._ The medium was substituted with a new medium containing twofold dilutions of PG (Sigma, China) before incubated overnight at 37^*o*^C with 5% CO_2_. Finally, 0.45% of MTT reagent in cell culture media was substituted and incubated for 4 h before adding 100 μL of 100× DMSO. The plate was read at 570 nm after 5 min using Versamax (molecular devices, United States). The rate of cell viability was calculated using the following formula:

$$\mathrm{Rate}\,\mathrm{of}\,\mathrm{viability}=\frac{\left(\mathrm{OD}\,\mathrm{compound}-\mathrm{OD}\,\mathrm{blank}\right)}{\left(\mathrm{OD}\,\mathrm{control}-\mathrm{OD}\,\mathrm{blank}\right)}\times 100$$

### Minimum Inhibitory Concentration and Minimum Bactericidal Concentration Determination

The minimum inhibitory concentration (MIC) of MAR and PG was done using the micro-broth dilution method as described by the Clinical Laboratory and Standard Institute (CLSI) (CLSI, 2017). A two-fold dilution of MAR and PG in Muller Hinton broth II (MHBII, Difco, BD, United States) and RPMI medium were added to 10^5^ CFU/mL of the three strains of *S*. Typhimurium. The bacteria were cultured for 24 h at 37^*o*^C aerobically and the results were read using a plate reader at 600 nm. To determine the minimum bactericidal concentration (MBC), 20 μL of the suspension from the microplate starting from the MIC onwards were taken and plated to LB agar. The plates were incubated at 37^*o*^C for 48 h to determine the possible slowly growing bacteria. The test was conducted three times in duplicate.

### Fractional Inhibitory Concentration Determination

The fractional inhibitory concentration (FIC) of the combination of MAR with PG was conducted using a checkerboard method (Leber, 2016). A final concentration of 10^5^ CFU/mL of the bacteria was added to the various fractional concentration of MAR and PG. The dilution was made in a 96-well plate and incubated aerobically overnight at 37^*o*^C. Finally, the results were read both visually and on a plate reader at 600 nm.

### Time-Kill Assay

A time-kill assay was done as previously described with minor modifications (Sendi et al., 2015). An overnight grown *Salmonella* Typhimurium was inoculated into a 5 mL LB broth and incubated for 4 h at 37^*o*^C to obtain the logarithmic growing phase of bacteria. The bacteria were added to MHBII containing various concentrations of MAR and PG to make a final bacterial concentration of 10^5^ CFU/mL. The bacteria were cultured at 37^*o*^C and sampled at 0, 0.5, 1, 2, 4, 8, 12, and 24 h and cultured on LB agar plates for 48 h after serially diluted. The results were read and recorded after a bacterial count.

### Bacterial Invasion and Intracellular Inhibition Assay

For bacterial invasion inhibition experiments, the gentamicin protection assay was performed as previously described with minor modifications (Wu et al., 2014). Briefly, PG (30 μg/mL) alone or with sub-MIC of MAR (0.015 μg/mL for susceptible and 0.25 μg/mL for MAR resistant bacteria) was added to the fully confluent Caco-2 cells and RAW 264.7 cells (10^5^ cells/mL) before incubating for 30 min. *Salmonella* Typhimurium (10^7^ CFU/mL) was added and incubated for another 45 min after centrifuged at 500 *g* for 5 min. Gentamicin (100 μg/mL) was added and incubated for 30 min before cells were lysed by 0.1% Triton^®^ x-100 (Sigma, United States) for 10 min. Cells were washed with PBS at least three-times in each step. The solution was serially diluted and cultured on a LB agar plate for a bacterial count after overnight incubation.

For intracellular killing experiments, RAW 264.7 cells (10^5^ cells/mL) were grown on 24 well plates. The cells were infected with *Salmonella* Typhimurium (10^7^ CFU/mL) and incubated for 1 h after centrifugation at 500 *g* for 5 min. Then the cells were washed, and PG (30 μg/mL) alone or combined with the sub-MIC of MAR were added and incubated for 1 h at 37°C and 5% CO_2_. Finally, the cells were treated with gentamicin (100 μg/mL) for 1 h and lysed in 0.1% Triton^®^ x-100 before serially diluted and incubated on an agar plate overnight at 37^*o*^C for a bacterial count.

### Confocal Microscopy

Caco-2 cells (10^5^ cells/mL) were cultured on 12 mm glass coverslips in 24-well plates as described Johnson and Criss (2013). The cells were prepared and treated as mentioned above for the bacterial intracellular inhibition assay. The cells were rinsed gently in 0.1 M 3-(N-morpholino) propanesulfonic acid (MOPS), pH 7.2, containing 1 mM MgCl_2_ (MOPS/MgCl_2_). The rinsing solution from the cells were aspirated, and 0.5 mL Live/Dead Staining Solution containing 5 μM SYTO9, 30 μM propidium iodide (IP) (Invitrogen, United States), and 0.1% saponin in MOPS/MgCl_2_ were added. The cells incubated for 15 min at room temperature in the dark and rinsed with MOPS/MgCl_2._ The coverslips were inverted face down onto glass slides, and images were acquired using a fluorescence microscope (Carl Zeiss, LSM700, IL, United States) with excitation of 488 and 555 nm and emission range of 515–555 nm and 560–600 nm for SYTO9 and propidium iodide, respectively. The thicknesses of the optical sections of the confocal microscopy were 12.2 and 13.2 μm for PI and SYTO-9 fluorescence, respectively. Non-treated cells were used as a negative control. Comparison was made upon counting of green fluorescence.

### Fluoroquinolone Accumulation in Intact Bacteria

The accumulation of fluoroquinolone in intact bacteria was determined as previously described (Vergalli et al., 2020) using ciprofloxacin due to its increased yield of fluorescent signal intensity. *Salmonella* Typhimurium was grown at 37°C in LB to mid-exponential phase. The bacterial suspension was centrifuged at 6,000 × *g* for 15 min and concentrated tenfold in 50 mM sodium phosphate buffer at pH 7 supplemented with 5 mM MgCl_2_ (NaPi–MgCl_2_ buffer) to obtain a density of 6 × 10^9^ CFU/mL. In glass culture tubes, 2.4 ml of the bacterial suspension was incubated for 5 min at 37°C with ciprofloxacin at 10 μg/mL, or 100 μg/mL (final volume 3 ml), in the absence or in the presence of PG and a positive control, the efflux inhibitor PAβN (Phenylalanine-arginine β-naphthylamide) used at 40 μg/mL. Bacterial suspensions incubated without antibiotics was used as negative controls. Suspensions (800 μl) were then transferred to 1,100 μL of NaPi–MgCl_2_ buffer and centrifuged at 9,000 × *g* for 5 min at 4°C and the pelleted bacteria was collected. After centrifugation, pellets corresponding to 800 μl of bacterial suspensions were lysed with 500 μl of 0.1M Glycin-HCl pH 3 overnight at room temperature. After a centrifugation for 15 min at 9,000 × *g* at 4°C, 400 μl of lysates were mixed with 600 μl of Glycin-HCl buffer, and emission spectra were measured with a spectrofluorimeter (F-2500 Fluorescence Spectrophotometer, Hitachi, Japan). Excitation/emission range wavelengths (nm) used for detection of ciprofloxacin fluorescence signal with spectrofluorimetry was 275/435–450.

### Total RNA Extraction and Virulence Gene Expression

For virulence gene expression, *Salmonella* Typhimurium was cultured in LB broth containing 3M of NaCl and incubated with different concentrations of PG and sub-MIC of MAR at 37^*o*^C for 13 h. The total RNA was extracted using Trizol^®^ (Ambion^®^, life technologies, United States) reagent. The RNA purity and concentration were determined using Nanodrop measurement. RT-PCR premix (pioneer, Korea) was used to synthesize bacterial cDNA by adding random hexamers. Quantification of RNA was performed on CFX96 Touch^TM^ real-time PCR detection systems (Biorad, United States) using IQ^TM^ SYBR^®^ Green supermix for real-time PCR (Biorad, Singapore). The qRT-PCR condition was set for 95^*o*^C for 3 min and 40 cycles of 95^*o*^C for 10 s, 58^*o*^C for 15 s, and 72^*o*^C for 30 s for *hilA, invF, sipB*, and *acrA* genes. The primers used in the study are listed in Table 1.

**TABLE 1:** Primers used to detect invasion virulence genes of *Salmonella* Typhimurium.

Target gene	Primers
*hilA*	5′-CGGAAGCTTATTTGCGCCATGCTGAGGTAG-3′ 5′-GCATGGATCCCCGCCGGCGAGATTGTG-3′
*invF*	5′-ACAGTGCTCGTTTACGACCTGAAT-3′ 5′-AGACGACTGGTACTGATCGATAAT-3′
*sipB*	5′-ACGCGCAAAGCCGAGGAAAC-3′ 5′-CCCGTCGCCGCCTTCAC-3′
*sdiA*	5′- TTACATTGGGATGACGTGCT-3′ 5′- AACTGCTACGGGAGAACGAT-3′
*acrA*	5′-CGCAGTACTATGTCGGTGAATTTACAGGCG-3′
	5′-CGCGGATCCGTCTTAACGGCTCCTGTTTAA-3′,
*rck*	5′-GTTGTATCCCGGCATGCTGAT-3′
	5′-ATATGCCCAGAGCCGGATAGAG-3′
*rrsG*	5′-GTTACCCGCAGAAGAAGCAC-3′
	5′- CACATCCGACTTGACAGACC 3′

### Quorum Sensing Gene Inhibition

To determine the effect of PG on the genetic expression of quorum sensing genes of *Salmonella* Typhimurium (Li et al., 2014), *N*-Acyl homoserine lactone (AHL, 1 μmol/mL) was added to the LB broth containing bacteria and incubated with 30, 100 μg/mL of PG and in combination with sub-MIC of MAR for 8 h. Furanone at 10 μg/mL (Sigma, St. Louis, MO, United States) was used as a positive control for quorum sensing inhibition. Whereas bacteria exposed to AHL without treatment with PG and MAR was used as a negative control. The suspension was centrifuged, and the pellet was processed for RNA extraction using Trizol reagent as described above. The qRT-PCR was set at a denaturation temperature of 95^*o*^C for 30 s, followed by 40 cycles of 95^*o*^C for 5 s, 60^*o*^C for 30 s, and dissociation of 95^*o*^C for 15 s and 60^*o*^C for 30 s.

### Prediction of Ligand-Protein Docking

The prediction of the binding and affinity of PG to the virulence and quorum sensing proteins of *Salmonella* Typhimurium was performed using iGEMDOCK (Version 2.1; NCTU, Hsinchu City, Taiwan) a graphical environment for recognizing pharmacological interactions and virtual screening (Yang and Chen, 2004). The chemical structure depiction for PG was retrieved from the PubChem database (Kim et al., 2019). Whereas the sequences of the bacterial virulence proteins were retrieved from the NCBI database. The protein structure was retrieved from PDB database (Berman et al., 2000; Burley et al., 2019) and predicted by RaptorX (Wang et al., 2017a,b, 2018; Xu, 2019; Xu and Wang, 2019). In addition, for the interactive visualization and analysis of molecular structures UCSF Chimera (Chimera, version 1.15, RBVI, San Francisco, CA, United States) was used (Pettersen et al., 2004).

### Statistical Analysis

Graphpad prism 7 (GraphPad Software, La Jolla, CA, United States) was used to analyze the data and one-way analysis of variance (ANOVA) followed by Tukey’s multiple comparison test was used to compare between treatment groups and compute the *p*-value. *P*-value was considered significant if *P* < 0.05.

## Results

**FIGURE 1:**
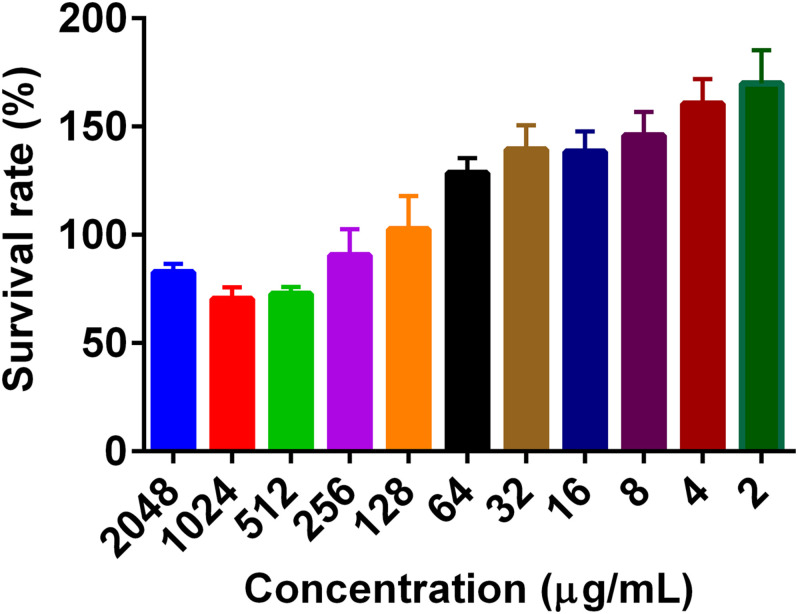
The effect of pyrogallol on the cell viability. The cells were treated with different concentrations of PG and incubated overnight, and the cell viability was determined using MTT reagent. Data is presented as Mean ± SEM.

To determine the effect of PG on mammalian cell growth, cell viability was investigated using the MTT reagent. PG at a concentration of 2,048 μg/mL did not show any significant cytotoxic activity on the viability of both Caco-2 and RAW 264.7 cells. The cell viability was more than 100% for concentrations less than 256 μg/mL (Figure 1).

The MIC and MBC of PG for all three strains were 128 and 256 μg/mL, respectively. The MIC of MAR against *Salmonella* Typhimurium ATTC 14028 and field isolate strain 2 (S-2) was 0.031 μg/mL, whereas it was 0.5 μg/mL for field isolate strain 15 (S-15). The MBC of MAR against ATTC 14028 and S-2 was 0.125 but 2 μg/mL for S-15. The log IC_50_ for MAR was −1.793, −0.5955, and −1.478, whereas it was 1.82, 1.459, and 1.511 for PG against ATCC 14028, S-15, and S-2 strains, respectively. The lowest FIC index results obtained for MAR in combination with PG in all the three strains were 0.5 (Table 2). Thus, for the consecutive experiments, we selected a concentration of 30 μg/mL of PG, which showed no antibacterial activity.

**TABLE 2:** MIC, MBC, and FIC_*index*_ of pyrogallol and marbofloxacin.

Strain	PG_MIC (μg/mL)	MAR_MIC (μg/mL)	PG_MBC (μg/mL)	MAR_MBC (μg/mL)	FIC_*index*_
S-2*	128	0.031	256	0.125	0.5
ATTC 14028	128	0.031	256	0.125	0.52
S-15**	128	0.5	256	2	0.75

*S-2 field strain of *Salmonella* Typhimurium susceptible to marbofloxacin. **S-15 field strain of *Salmonella* Typhimurium resistant to marbofloxacin. PG, pyrogallol; MAR, marbofloxacin; MIC, minimum inhibitory concentration; MBC, minimum bactericidal concentration; FIC, fractional inhibitory concentration.

**FIGURE 2:**
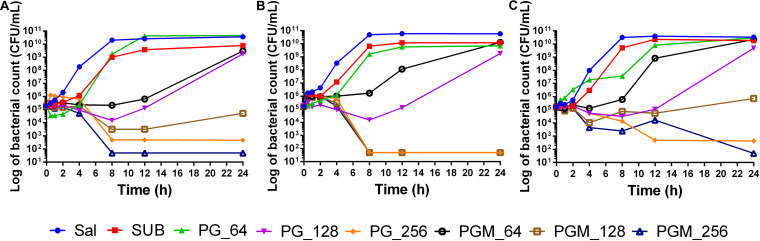
Time-Kill assay of pyrogallol alone and in combination with sub-MIC of MAR against different strains of *Salmonella* Typhimurium. **(A)** ATCC 14028 **(B)** susceptible field isolate (S-2), and **(C)** intermediately resistant field isolate (S-15). Sal: non-treated *Salmonella* Typhimurium control; SUB: treated with sub-MIC of marbofloxacin alone; PG-64: treated with only pyrogallol (64 μg/mL); PG-128: treated with only pyrogallol (128 μg/mL); PG-256: treated with only pyrogallol (256 μg/mL); PGM_64: treated with a combination of pyrogallol 64 μg/mL and sub-MIC of marbofloxacin; PGM_128: treated with a combination of pyrogallol 128 μg/mL and sub-MIC of marbofloxacin; PGM_256: treated with a combination of pyrogallol 256 μg/mL and sub-MIC of marbofloxacin.

The time-kill assay was performed to detect the combined effect of sub_MIC of MAR with different concentrations of PG in a dynamic model. The test was conducted for 24 h. The sub-MIC of MAR in combination with 128 μg/mL of PG reduce *Salmonella* Typhimurium (ATCC 14028) CFU/mL by 2-log within 8 h. Whereas, the 2-log reduction for the S-2 strain was achieved after 4 h of incubation at all tested concentrations of PG except the 64 μg/mL. The 2-log reduction for the S-15 strain was observed after 12 h of incubation by the same concentration of PG and MAR (Figure 2). In strain ATCC 14028 and S-15, the combination of PG (256 μg/mL) and sub-MIC of MAR showed early killing of the bacteria in comparison with PG (256 μg/mL) used alone. However, PG (64 and 128 μg/mL) alone did not show to inhibit the growth of *Salmonella* Typhimurium strains tested.

**FIGURE 3:**
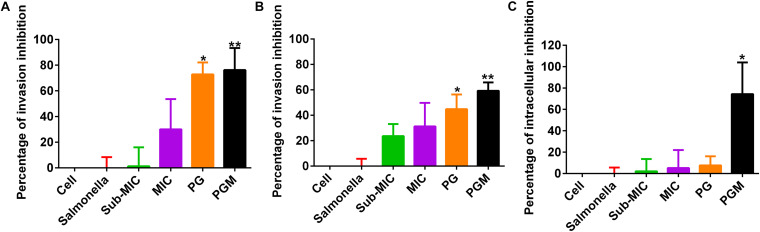
Percentage of inhibition of *Salmonella* Typhimurium invasion and intracellular survival by pyrogallol in Caco-2 and RAW 264.7 cells. **(A)** Inhibition of *Salmonella* Typhimurium invasion in Caco-2 cells; **(B)** inhibition of *Salmonella* Typhimurium invasion in RAW 264.7 cells and **(C)** Inhibition of intracellular survival of *Salmonella* Typhimurium by pyrogallol in RAW 246.7 cells. Cell – non-infected cells; Sal – infected with *Salmonella* Typhimurium but not treated; sub-MIC – infected with *Salmonella* Typhimurium and treated with sub-MIC of MAR alone; MIC – infected with *Salmonella* Typhimurium and treated with the MIC of MAR alone; PG – infected with *Salmonella* Typhimurium and treated with 30 μg/mL of PG; PGM – infected with *Salmonella* Typhimurium and treated with a combination of 30 μg/mL of PG and sub-MIC of MAR; *P < 0.05 and **P < 0.01.

For the bacterial invasion inhibition assay, PG at a concentration of 30 μg/mL alone and in combination with sub-MIC of MAR was used. PG (30 μg/mL) inhibited 72.75 and 44.8% of *Salmonella* Typhimurium invasion in Caco-2 and Raw 264.7 cells, respectively. Whereas, for the combination of PG with sub-MIC of MAR, the inhibition percentage was increased to 76.19 and 59.3% in Caco-2 and Raw 264.7 cells, respectively. A significant difference (*P* < 0.05) was observed in the inhibition of *Salmonella* Typhimurium invasion in both cell lines by PG alone and combination with MAR. However, no significant difference was observed between Caco-2 and Raw 264.7 cells (Figures 3A,B).

Likewise, PG (30 μg/mL) inhibited 7.69% of intracellular *Salmonella* Typhimurium in RAW264.7 cells. Whereas in combination with sub-MIC of MAR, the suppression of the intracellular survival of *Salmonella* Typhimurium increased to 74.36%. A significant difference (*P* < 0.05) was indicated in cells treated with the combination of PG with sub-MIC of MAR (Figure 3C).

**FIGURE 4:**
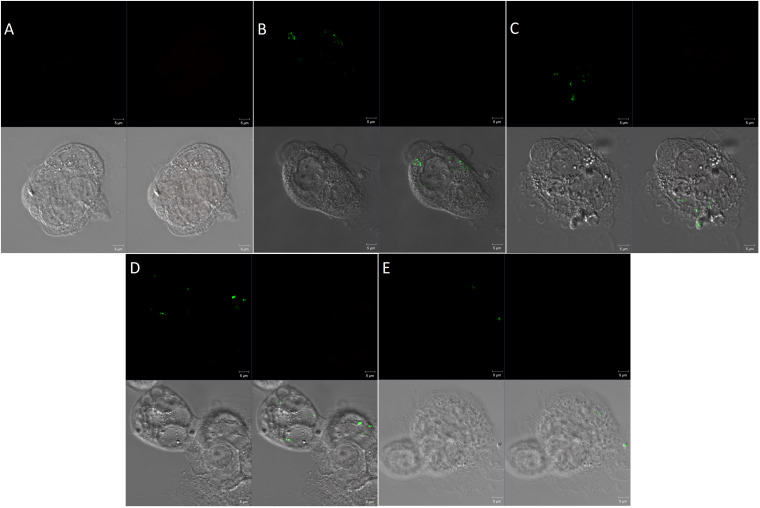
Confocal microscopy of invasion of Caco-2 cells by *Salmonella* Typhimurium **(A)** Uninfected cells, **(B)** cells infected with *Salmonella* Typhimurium but not treated, **(C)** Infected cells and treated with sub_MIC of MAR. **(D)** Cells infected and treated with pyrogallol (30 μg/mL) and **(E)** cells infected and treated with pyrogallol and sub-MIC of MAR. The top left panel is a SYTO-9 fluorescence; the top right panel is a propidium iodide fluorescence; the bottom left panel indicates cell structure without fluorescent; the bottom right panel shows a merged image. (Red, dead bacteria; Green, live bacteria; image: ×1,000 magnification).

In compliance with the above findings, the confocal microscopy had also revealed the percentage of intracellular bacteria was significantly suppressed by the activity of PG alone and its combination with sub-MIC of MAR (Figure 4).

**FIGURE 5:**
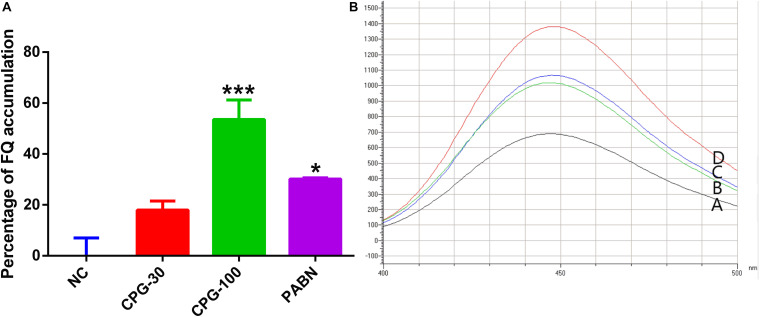
Accumulation of fluoroquinolone by pyrogallol. **(A)** The graph showed the increase in the accumulation of ciprofloxacin by various concentration of pyrogallol using PAβN as a positive control. **(B)** Fluorescence microscopy showed increased accumulation of fluoroquinolone by pyrogallol (A, Ciprofloxacin; B, Ciprofloxacin with PG 30 μg/mL; C, Ciprofloxacin with 100 μg/mL pyrogallol; D, Ciprofloxacin with 40 μg/mL of PAβN). NC – bacteria exposed to ciprofloxacin only; CPG-30 – bacteria exposed to ciprofloxacin and 30 μg/mL of pyrogallol; CPG-100 – bacteria exposed to ciprofloxacin and 100 μg/mL of pyrogallol; PAβN – bacteria exposed to ciprofloxacin and 40 μg/mL of PAβN. *p < 0.05.

The fluoroquinolone accumulation assay indicated that PG (30 μg/mL) increased the accumulation of ciprofloxacin by 15.2%. The accumulation was increased to 34.9% upon increasing the concentration of PG to 100 μg/mL which showed a significant difference in comparison with the negative control (Figure 5).

**FIGURE 6:**
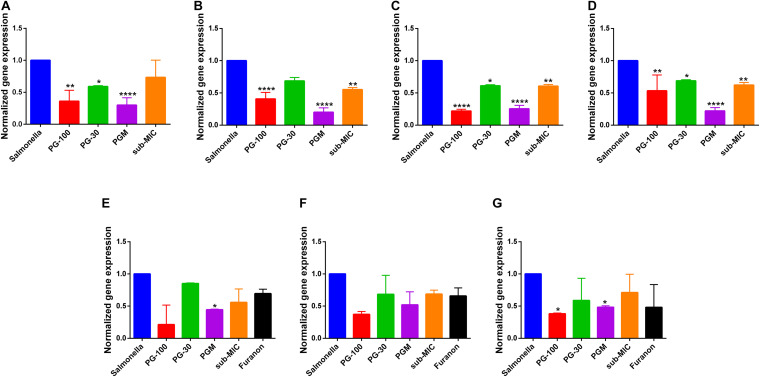
Effect of pyrogallol on the expression of Salmonella invasion and quorum sensing virulence genes. **(A)** acrA **(B)** hilA **(C)** invF, **(D)** sipB, **(E)** sdiA **(F)** srgE, and **(G)** rck gene. Salmonella – untreated culture; PG-100 – treated with 100 μg/mL of pyrogallol; PG-30 – treated with 30 μg/mL of pyrogallol; PGM – treated with a combination of 30 μg/mL of pyrogallol and sub-MIC of marbofloxacin; sub-MIC – treated with sub-MIC of marbofloxacin alone. For the quorum sensing gene expression assay all groups are treated with N-Acyl homoserine lactone (AHL, 1 μmol/mL). *P < 0.05; **p < 0.01; ****P < 0.001.

The genetic expression level of different virulent invasion genes of *Salmonella* Typhimurium was evaluated using qRT-PCR. The result indicated that bacteria treated with PG (30 μg/mL) have a reduction of gene expression of *hilA, invF*, *sipB*, and *acrA* genes by 14.6, 15.4, 13.6, and 36%, respectively. The suppression of the gene expression level was dose dependent. At a higher concentration of PG (100 μg/mL), the inhibition was increased significantly to 59.3, 78.1, 46.7, and 63.8% for *hilA, invF*, *sipB*, and *acrA* genes, respectively. Whereas the combination of PG with sub-MIC of MAR showed a significant difference in suppressing the expression of *hilA, invF*, *sipB*, and *acrA* genes by 80, 74.6, 78, and 70.1%, respectively (Figures 6A–D).

The effect of PG on the quorum sensing genes of *Salmonella* Typhimurium was evaluated using AHL as an inducer of quorum sensing signaling. The genetic expression levels of *sdiA, srgE*, and *rck* genes were reduced by 14.9, 31.6, and 41.2% upon treatment with PG (30 μg/mL), respectively. However, the inhibition was increased to 78.6, 62.8, and 61.8% for a higher concentration of PG (100 μg/mL), respectively. Whereas the combination of PG (30 μg/mL) with the sub-MIC of MAR inhibited the expression level of *sdiA, srgE*, and *rck* genes by 55.5, 48.1, and 46.7%, respectively (Figures 6E–G).

**FIGURE 7:**
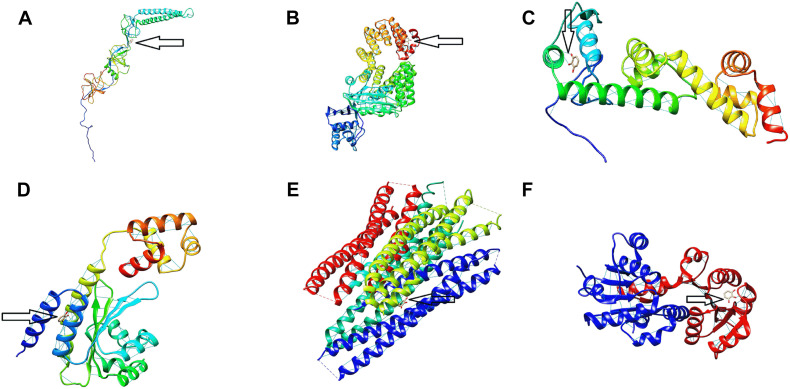
Protein binding site of pyrogallol on **(A)** acrA, **(B)** hilA **(C)** invF, **(D)** sdiA, **(E)** sipB, and **(F)** rck proteins of *Salmonella* Typhimurium. The arrow indicates the binding site of pyrogallol with the respective proteins and the hydrogen-bond formed between the protein and ligand.

The binding energy affinity of PG to the suppressed proteins of acrA, hilA, invF, sdiA, sipB, and rck, proteins of *Salmonella* Typhimurium is presented in Table 3. The binding of PG with the amino acid residues showed a hydrogen bond linkage for of acrA, hilA, invF, sdiA, and rck, proteins (Figure 7).

**TABLE 3:** Binding affinity of pyrogallol to *Salmonella* Typhimurium virulence proteins.

Proteins	Binding energy (Kcal)	Van der waals	Hydrogen bond
acrA	−67.2353	−41.2766	−25.9587
hilA	−72.176	−62.6	−9.57602
invF	−70.1798	−56.8533	−13.3265
sdiA	−71.2049	−66.4945	−4.7104
rck	−73.9291	−47.754	−26.1751
sipB	−49.8305	−43.3426	−6.48786

## Discussion

Treatment of intracellular bacteria is a major health challenge globally. Besides, these bacteria developed strategies to evade the host defense mechanism, invade the cells and reside intracellularly. Moreover, regardless of the possibility of developing new antimicrobials, microbial agents acquire resistance to various drugs (Hrvatin, 2017). Even though quinolones are referred to as the therapeutic options against intracellular pathogens, multiple bacteria are developing resistance against these conserved groups of antimicrobial agents (Van Bambeke et al., 2005). Similarly, *Salmonella* Typhimurium developed resistance against fluoroquinolones (de Jong et al., 2012). Hence, searching for an active compound that targets bacterial invasion and intracellular survival has paramount significance. In consequence, combination therapies are emerging as an alternative for the treatment of intracellular residing and multidrug-resistant microbes (Worthington and Melander, 2013).

Polyphenols are characterized by the presence of multiple hydroxyl groups in their benzene ring and are known for their antioxidant properties and modulation of enzymatic and cell receptors (Middleton et al., 2000). PG, a polyphenol, possesses antioxidant, antimicrobial activity, quorum sensing inhibition, and suppression of cytotoxicity properties (Ni et al., 2008; Baruah et al., 2015; Mendes et al., 2015). Therefore, in this study, we discovered the effect of PG alone or in combination with MAR in the invasion and intracellular survival of *Salmonella* Typhimurium isolated from pigs.

The determination of cytotoxicity is critical in detecting the potential toxic effect of a given compound in mammalian cells. In our findings, PG did not show any cytotoxic activity against both Caco-2 and Raw 264.7 cells even at a more significant concentration. In agreement with this, PG also did not show any cytotoxicity on the growth of HeLa cells (Lim et al., 2016).

The anti-infective activity of polyphenols has been described in various bacterial and fungal agents (Yanagawa et al., 2003; Friedman et al., 2006). They have indicated strong inhibitory activity against both Gram-negative and positive bacteria. In this study, we have indicated the antibacterial activity of PG against two field strains of *Salmonella* Typhimurium isolated from a clinical infection of pigs and ATCC control strains. In all the cases, PG exhibited similar antibacterial activity against *Salmonella Typhimurium* isolates at the same concentration. In the same way, PG has shown antibacterial activity against *Pseudomonas putida, P. pyocyanea, Corynebacterium xerosis, Vibrio parahaemolyticus, V. vulnificus*, and *Staphylococcus aureus* (Kocaçalişkan et al., 2006; Lim et al., 2016; Lima et al., 2016; Tinh et al., 2016). This indicated the likelihood of PG in the making of a novel antibacterial agent against infectious agents (Lim et al., 2016).

Moreover, in combination with the sub-MIC of MAR, which remains a widely used drug in veterinary medicine, PG resulted in the most substantial effect and presented self-induced synergism. Our findings agree with previous reports that PG has shown a synergistic effect with norfloxacin and gentamicin against *S. aureus* (Lima et al., 2016). Currently, there is a shortage of newly discovered antibiotics against multidrug-resistant bacteria and to worsen the situation, bacteria, particularly Gram-negative bacteria, develop resistance to most of the currently available antimicrobials (Lee et al., 2009; Freire-Moran et al., 2011). Thus, to alleviate this challenge, a combination of antimicrobial agents with other antimicrobials or other compounds is critical (Worthington and Melander, 2013). The potentiation effect of PG delivers a significant contribution to overcoming antimicrobial resistance. Hence, the antimicrobial activity observed by PG could have a significant contribution to reducing drug resistance and become an alternative to the currently available drugs (Worthington and Melander, 2013).

For successful treatment of intracellular bacteria, once the antimicrobial agents traverse the host cell membrane, they must be maintained and amass at adequate concentrations for a specified time (Kamaruzzaman et al., 2017). The accumulation of a drug is dependent on the suppression or expression of acrAB (Vergalli et al., 2017). PG increased the accumulation of fluoroquinolone in intact *Salmonella* Typhimurium cells. The presence of a higher concentration of fluoroquinolone suggests the inhibition of an active efflux pump (Okusu et al., 1996). This could be due to the downregulation of an efflux inhibitor acrA genes by PG. This indeed will increase the effectiveness of the drug in killing intracellular bacteria.

In addition to its antibacterial activity, the *in vitro* cell culture experiments demonstrated the potential of PG to inhibit *Salmonella* invasion and its intracellular survival. At the lowest concentration (30 μg/mL), four times less than its MIC, specifically in combination with sub-MIC of MAR, PG inhibited about 70% of *Salmonella* Typhimurium invasion in epithelial cells and intracellular survival in the macrophages. Methyl gallate, which is a polyphenol also inhibited bacterial adhesion, invasion and intracellular survival (Birhanu et al., 2018). This also agrees with the works of Tsou et al. (2016) who described the inhibition of *Salmonella Typhimurium* invasion by flavonoids like baicalein and quercetin. This inhibition targeted the SPI-1 T3SS substrates of the bacteria. This more considerably extends the role of PG in the production of a potent antimicrobial agent.

It is indicated that PG has downregulated the genetic expression of *hilA* and *invF* genes, critical for invading host cells and entering their cytoplasm. *Salmonella* Typhimurium activates the SPI-1 T3SS for entering the intestinal epithelium cells. HilA is the central regulator of invasion of epithelial cells and a transcriptional activator of SPI-1 genes including *invF* (Penheiter et al., 1997; Ellis et al., 2017). The most common mechanisms of inhibition by polyphenols are downregulating the genetic expression of virulence factors, quorum sensing signal inhibition, biofilm formation, and decreasing bacterial swarm motility (Kang-Mu et al., 2009; Daglia, 2012; Hossain et al., 2017; Birhanu et al., 2018). *InvF* is essential for the expression of the SPI-1 gene, which encodes effector proteins and their translocation into the cytosol (Darwin and Miller, 2000). Hence, the downregulation of these genes by PG could suppress the activity of other SPI-1 genes of *Salmonella* Typhimurium involved in cell invasion and intracellular survival and leads to the killing of the bacteria.

Furthermore, PG suppressed the expression of the *sipB gene*, which is critical for encoding and translocating other SPI-1 T3SS *Salmonella* Typhimurium secreted effector proteins and is essential for mammalian cell invasion (Hayward et al., 2000). Furthermore, sipB expedite adherance of *Salmonella* Typhimurium to target host cells (Lara-Tejero and Galán, 2009). Hence, the downregulation of *sipB* by PG might play a significant role in decreasing the invasion and intracellular bacteria.

PG also suppressed the level of expression of *Salmonella* Typhimurium *acrA* gene which is a resistance nodulation cell division gene (Guérin et al., 2016). It encodes for a membrane fusion protein, which is an efflux pump responsible for decreasing intracellular accumulation of drugs and results in antimicrobial resistance (Nishino et al., 2006; Yamasaki et al., 2011). The downregulation of *acrA* by PG will further confirm its paramount importance in preventing antimicrobial resistance and could be considered as an efflux pump inhibitor.

Furthermore, PG suppressed the quorum sensing related genes of *Salmonella*. *SdiA*, a LuxR homolog, is a quorum-sensing signal detector and regulator in *Salmonella Typhimurium* and activates *srgE* and *rck* genes (Dyszel et al., 2010; Li et al., 2014). Rck confers adhesiveness and invasiveness to intestinal epithelial cells (Cirillo et al., 1996; Michael et al., 2001). In addition, *rck* mediates the zipper-like internalization of *Salmonella* into the host cells (Rosselin et al., 2010). This further signifies the potential of PG in inhibiting bacterial invasion and combat clinical infections.

The decreased energy value of the protein-ligand interaction showed the high binding affinity of PG to the virulence and quorum sensing proteins of *Salmonella* Typhimurium. Although PG did not show to totally block the expression of virulence genes, it could have the potential to bind the remaining translated *Salmonella* effector proteins. This also indicates the likely chance of interfering with their activity and limiting the pathogenicity of the bacteria. Which further inhibits its invasion and intracellular survival.

## Conclusion

In conclusion, in this study, we have indicated the antimicrobial and anti-invasive activity of PG against *Salmonella* Typhimurium in vitro study. PG is effective in killing intracellular bacteria alone and in combination with MAR. Furthermore, it increased the accumulation of fluoroquinolones. PG suppresses the virulence and quorum sensing genes of *Salmonella* Typhimurium, which are critical for invasion and intracellular survival of the bacteria and subsequently establish infection in the host cells. PG showed a self-induced potentiation effect to the most conserved drug of veterinary importance, MAR, in which certain bacterial agents are currently developing resistance against it. This suggests PG could be a potential efflux pump inhibitor and a candidate as an antimicrobial agent to prevent bacterial invasion, its intracellular survival, and antimicrobial resistance. However, further *in vivo* pharmacokinetic and pharmacodynamic studies should be conducted for comprehensive understanding before its preclinical trial.

## Data Availability Statement

The original contributions presented in the study are included in the article/supplementary material, further inquiries can be directed to the corresponding author/s.

## Author Contributions

BB designed and carried out the experiment, analyzed the results, and wrote the manuscript. E-BL analyzed the results and reviewed the manuscript. S-JL and S-CP designed the study and revised the manuscript. All authors read and approved the final draft.

## Conflict of Interest

The authors declare that the research was conducted in the absence of any commercial or financial relationships that could be construed as a potential conflict of interest.

## References

[B1] Agbor T. A. Mccormick B. A. (2011). *Salmonella* effectors: Important players modulating host cell function during infection. *Cell. Microbiol.* 13 1858–1869. 10.1111/j.1462-5822.2011.01701.x 21902796 PMC3381885

[B2] Baruah K. Duy Phong H. P. P. Norouzitallab P. Defoirdt T. Bossier P. (2015). The gnotobiotic brine shrimp (*Artemia franciscana*) model system reveals that the phenolic compound pyrogallol protects against infection through its prooxidant activity. *Free Radic. Biol. Med.* 89 593–601. 10.1016/j.freeradbiomed.2015.10.397 26459033

[B3] Berman H. M. Westbrook J. Feng Z. Gilliland G. Bhat T. N. Weissig H. (2000). The protein data bank. *Nucleic Acids Res.* 28 235–242. 10592235 10.1093/nar/28.1.235 PMC102472

[B4] Birhanu B. T. Park N. H. Lee S. J. Hossain M. A. Park S. C. (2018). Inhibition of *Salmonella* Typhimurium adhesion, invasion, and intracellular survival via treatment with methyl gallate alone and in combination with marbofloxacin. *Vet Res.* 49:101. 10.1186/s13567-018-0597-8 PMC6389159 30286813

[B5] Burley S. K. Berman H. M. Bhikadiya C. Bi C. Chen L. Di Costanzo L. (2019). RCSB Protein Data Bank: Biological macromolecular structures enabling research and education in fundamental biology, biomedicine, biotechnology and energy. *Nucleic Acids Res.* 47 D464–D474. 30357411 10.1093/nar/gky1004 PMC6324064

[B6] Cirillo D. M. Heffernan E. J. Wu L. Harwood J. Fierer J. Guiney D. G. (1996). Identification of a domain in Rck, a product of the *Salmonella* typhimurium virulence plasmid, required for both serum resistance and cell invasion. *Infect. Immun.* 64 2019–2023. 10.1128/iai.64.6.2019-2023.1996 8675302 PMC174031

[B7] CLSI (2017). *M100-S23 Performance Standards for Antimicrobial Susceptibility Testing; Twenty-Third Informational Supplement An informational supplement for global application developed through the Clinical and Laboratory Standards Institute consensus process*, 27 Edn, eds Patel J. Weinstein M. George E. Jenkins S. Lewis J. Limbago B. (Wayne, PA: Clinical and Laboratory Standards Institute).

[B8] Daglia M. (2012). Polyphenols as antimicrobial agents. *Curr. Opin. Biotechnol.* 23 174–181. 10.1016/j.copbio.2011.08.007 21925860

[B9] Darwin K. H. Miller V. L. (2000). The putative invasion protein chaperone SicA acts together with InvF to activate the expression of *Salmonella* typhimurium virulence genes. *Mol. Microbiol.* 35 949–960. 10.1046/j.1365-2958.2000.01772.x 10692170

[B10] de Jong A. Stephan B. Silley P. (2012). Fluoroquinolone resistance of *Escherichia coli* and *Salmonella* from healthy livestock and poultry in the EU. *J. Appl. Microbiol.* 112 239–245. 10.1111/j.1365-2672.2011.05193.x 22066763

[B11] Dyszel J. L. Smith J. N. Lucas D. E. Soares J. A. Swearingen M. C. Vross M. A. (2010). *Salmonella enterica* serovar typhimurium can detect acyl homoserine lactone production by Yersinia enterocolitica in mice. *J. Bacteriol.* 192 29–37. 10.1128/jb.01139-09 19820103 PMC2798265

[B12] Ellis M. J. Trussler R. S. Charles O. Haniford D. B. (2017). A transposon-derived small RNA regulates gene expression in *Salmonella* Typhimurium. *Nucleic Acids Res.* 45 5470–5486. 10.1093/nar/gkx094 28335027 PMC5435999

[B13] Fischbach M. A. (2011). Combination therapies for combating antimicrobial resistance. *Curr. Opin. Microbiol.* 14 519–523. 10.1016/j.mib.2011.08.003 21900036 PMC3196371

[B14] Freire-Moran L. Aronsson B. Manz C. Gyssens I. C. So A. D. Monnet D. L. (2011). Critical shortage of new antibiotics in development against multidrug-resistant bacteria – Time to react is now. *Drug Resist Updat.* 2011 118–124. 10.1016/j.drup.2011.02.003 21435939

[B15] Friedman M. Henika P. R. Levin C. E. Mandrell R. E. Kozukue N. (2006). Antimicrobial activities of tea catechins and theaflavins and tea extracts against Bacillus cereus. *J. Food Protect.* 2006 354–361. 10.4315/0362-028x-69.2.354 16496576

[B16] Guérin F. Lallement C. Isnard C. Dhalluin A. Cattoir V. Giard J. C. (2016). Landscape of resistance-nodulation-cell division (RND)-type efflux pumps in *Enterobacter cloacae* complex. *Antimicrob Agents Chemother.* 60 2373–2382. 10.1128/aac.02840-15 26856831 PMC4808149

[B17] Haraga A. Ohlson M. B. Miller S. I. (2008). *Salmonella*e interplay with host cells. *Nat. Rev. Microbiol.* 6 53–66. 18026123 10.1038/nrmicro1788

[B18] Hayward R. D. McGhie E. J. Koronakis V. (2000). Membrane fusion activity of purified SipB, a *Salmonella* surface protein essential for mammalian cell invasion. *Mol. Microbiol.* 37 727–739. 10.1046/j.1365-2958.2000.02027.x 10972796

[B19] Hossain M. A. Lee S. J. Park N. H. Mechesso A. F. Birhanu B. T. Kang J. (2017). Impact of phenolic compounds in the acyl homoserine lactone-mediated quorum sensing regulatory pathways. *Sci. Rep.* 7 1–16. 28878346 10.1038/s41598-017-10997-5 PMC5587592

[B20] Hrvatin V. (2017). Combating antibiotic resistance: New drugs or alternative therapies? *CMAJ*? 189:E1199 10.1002/9783527622931.ch1 PMC5602506 28923803

[B21] Johnson M. B. Criss A. K. (2013). Fluorescence microscopy methods for determining the viability of bacteria in association with mammalian cells. *J. Vis. Exp.* 50729. 10.3791/50729 24056524 PMC3814296

[B22] Kamaruzzaman N. F. Kendall S. Good L. (2017). Targeting the hard to reach: challenges and novel strategies in the treatment of intracellular bacterial infections. *Br. J. Pharmacol.* 174 2225–2236. 10.1111/bph.13664 27925153 PMC5481648

[B23] Kang-Mu L. E. E. Wan-Seok K. I. M. Jeesun L. I. M. Sunyoung N. A. M. Youn M. I. N. Seong-Won N. A. M. (2009). Antipathogenic properties of green tea polyphenol epigallocatechin gallate at concentrations below the MIC against enterohemorrhagic *escherichia coli* O157:H7. *J. Food Prot.* 72 325–331. 10.4315/0362-028x-72.2.325 19350976

[B24] Kim S. Chen J. Cheng T. Gindulyte A. He J. He S. (2019). PubChem 2019 update: Improved access to chemical data. *Nucleic Acids Res.* 47 D1102–D1109. 30371825 10.1093/nar/gky1033 PMC6324075

[B25] Kocaçalişkan I. Talan I. Terzi I. (2006). Antimicrobial activity of catechol and pyrogallol as allelochemicals. *Zeitschr. Naturf. Sect. C J. Biosci.* 61 639–642. 10.1515/znc-2006-9-1004 17137106

[B26] Lara-Tejero M. Galán J. E. (2009). *Salmonella enterica* serovar typhimurium pathogenicity island 1-encoded type III secretion system translocases mediate intimate attachment to nonphagocytic cells. *Infect Immun.* 77 2635–2642. 10.1128/iai.00077-09 19364837 PMC2708559

[B27] Laupland K. B. Schønheyder H. C. Kennedy K. J. Lyytikäinen O. Valiquette L. Galbraith J. (2010). *Salmonella enterica* bacteraemia: a multi-national population-based cohort study. *BMC Infect Dis.* 10:95. 10.1186/1471-2334-10-95 PMC2861061 20398281

[B28] Leber A. L. (2016). *Clinical Microbiology Procedures Handbook*, 4 Edn, ed. Leber A. L. (Columbus, OH: ASM Press).

[B29] Lee J. H. Jeong S. H. Cha S.-S. Lee S. H. (2009). New Disturbing trend in antimicrobial resistance of gram-negative pathogens. *PLoS Pathog.* 5:e1000221. 10.1371/journal.ppat.1000221 19325878 PMC2654509

[B30] Lee S. J. Park N. H. Mechesso A. F. Lee K. J. Park S. C. (2017). The phenotypic and molecular resistance induced by a single-exposure to sub-mutant prevention concentration of marbofloxacin in *Salmonella typhimurium* isolates from swine. *Vet. Microbiol.* 207 29–35. 10.1016/j.vetmic.2017.05.026 28757036

[B31] Li G. Yan C. Xu Y. Feng Y. Wu Q. Lv X. (2014). Punicalagin inhibits *Salmonella* virulence factors and has anti-quorum-sensing potential. *Appl. Environ. Microbiol.* 80 6204–6211. 10.1128/aem.01458-14 25085489 PMC4178673

[B32] Lim J. Y. Kim C.-M. Rhee J. H. Kim Y. R. (2016). Effects of pyrogallol on growth and cytotoxicity of wild-type and katg mutant strains of vibrio vulnificus. *PLoS One* 11:e0167699. 10.1371/journal.pone.0167699 27936080 PMC5147952

[B33] Lima V. N. Oliveira-Tintino C. D. M. Santos E. S. Morais L. P. Tintino S. R. Freitas T. S. (2016). Antimicrobial and enhancement of the antibiotic activity by phenolic compounds: Gallic acid, caffeic acid and pyrogallol. *Microb. Pathog.* 99 56–61. 10.1016/j.micpath.2016.08.004 27497894

[B34] Liu Y. Jia Y. Yang K. Wang Z. (2020). Heterogeneous strategies to eliminate intracellular bacterial pathogens. *Front. Microbiol.* 11:563. 10.3389/fmicb.2020.00563 PMC7192003 32390959

[B35] Manach C. Scalbert A. Morand C. Rémésy C. Jiménez L. (2004). Polyphenols: food sources and bioavailability. *Am. J. Clin. Nutr.* 79 727–747. 10.1093/ajcn/79.5.727 15113710

[B36] Mason T. L. Bruce P. W. (1987). Inactivation of red beet β-glucan synthase by native and oxidized phenolic compounds. *Phytochemistry* 26 2197–2202. 10.1016/s0031-9422(00)84683-x

[B37] Mendes V. Vilaça R. De Freitas V. Ferreira P. M. Mateus N. Costa V. (2015). Effect of myricetin, pyrogallol, and phloroglucinol on yeast resistance to oxidative stress. *Oxid Med. Cell Longev.* 2015:782504. 10.1155/2015/782504 PMC4427115 26000072

[B38] Michael B. Smith J. N. Swift S. Heffron F. Ahmer B. M. M. (2001). SdiA of *Salmonella enterica* is a LuxR homolog that detects mixed microbial communities. *J. Bacteriol.* 183 5733–5742. 10.1128/jb.183.19.5733-5742.2001 11544237 PMC95466

[B39] Middleton E. Kandaswami C. Theoharides T. (2000). The effects of plant flavonoids on mammalian cells: implications for inflammation, heart disease, and cancer. *Pharmacol. Rev.* 52 673–751. 11121513

[B40] Moyle C. W. A. Cerezo A. B. Winterbone M. S. Hollands W. J. Alexeev Y. Needs P. W. (2015). Potent inhibition of VEGFR-2 activation by tight binding of green tea epigallocatechin gallate and apple procyanidins to VEGF: Relevance to angiogenesis. *Mol. Nutr. Food Res.* 59 401–412. 10.1002/mnfr.201400478 25546248 PMC4681316

[B41] Ni N. Choudhary G. Li M. Wang B. (2008). Pyrogallol and its analogs can antagonize bacterial quorum sensing in Vibrio harveyi. *Bioorganic. Med. Chem. Lett.* 18 1567–1572. 10.1016/j.bmcl.2008.01.081 18262415

[B42] Nishino K. Latifi T. Groisman E. A. (2006). Virulence and drug resistance roles of multidrug efflux systems of *Salmonella enterica* serovar *typhimurium*. *Mol. Microbiol.* 59 126–141. 10.1111/j.1365-2958.2005.04940.x 16359323

[B43] Okusu H. Ma D. Nikaido H. (1996). AcrAB efflux pump plays a major role in the antibiotic resistance phenotype of *Escherichia coli* multiple-antibiotic-resistance (Mar) mutants. *J. Bacteriol.* 178 306–308. 10.1128/jb.178.1.306-308.1996 8550435 PMC177656

[B44] Ozturk Sarikaya S. B. (2015). Acethylcholinesterase inhibitory potential and antioxidant properties of pyrogallol. *J. Enzyme Inhib. Med. Chem.* 30 761–766. 10.3109/14756366.2014.965700 25297710

[B45] Pandey K. B. Rizvi S. I. (2009). Plant polyphenols as dietary antioxidants in human health and disease. *Oxidat. Med. Cell. Long.* 2 270–278. 10.4161/oxim.2.5.9498 20716914 PMC2835915

[B46] Patel J. C. Galán J. E. (2005). Manipulation of the host actin cytoskeleton by *Salmonella* – All in the name of entry. *Curr. Opin. Microbiol.* 8 10–15. 10.1016/j.mib.2004.09.001 15694851

[B47] Penheiter K. L. Mathur N. Giles D. Fahlen T. Jones B. D. (1997). Non-invasive *Salmonella typhimurium* mutants are avirulent because of an inability to enter and destroy M cells of ileal Peyer’s patches. *Mol. Microbiol.* 24 697–709. 10.1046/j.1365-2958.1997.3741745.x 9194698

[B48] Pettersen E. F. Goddard T. D. Huang C. C. Couch G. S. Greenblatt D. M. Meng E. C. (2004). UCSF Chimera – A visualization system for exploratory research and analysis. *J. Comput. Chem.* 25 1605–1612. 10.1002/jcc.20084 15264254

[B49] Ribet D. Cossart P. (2015). How bacterial pathogens colonize their hosts and invade deeper tissues. *Microbes Infect.* 17 173–183. 10.1016/j.micinf.2015.01.004 25637951

[B50] Rosselin M. Virlogeux-Payant I. Roy C. Bottreau E. Sizaret P. Y. Mijouin L. (2010). Rck of *Salmonella enterica*, subspecies enterica serovar Enteritidis, mediates Zipper-like internalization. *Cell Res.* 20 647–664. 10.1038/cr.2010.45 20368731

[B51] Sendi P. Ruppen C. Sendi P. (2015). Time kill assays for *Streptococcus agalactiae* and synergy testing. *Protoc. Exch.* 28 126.

[B52] Taguri T. Tanaka T. Kouno I. (2006). Antibacterial spectrum of plant polyphenols and extracts depending upon hydroxyphenyl structure. *Biol. Pharm. Bull.* 29 2226–2235. 10.1248/bpb.29.2226 17077519

[B53] Thiennimitr P. Winter S. E. Bäumler A. J. (2012). *Salmonella*, the host and its microbiota. *Curr. Opin. Microbiol.* 12 108–114. 10.1016/j.mib.2011.10.002 22030447 PMC3265626

[B54] Tinh T. H. Nuidate T. Vuddhakul V. Rodkhum C. (2016). Antibacterial activity of pyrogallol, a polyphenol compound against vibrio parahaemolyticus isolated from the central region of thailand. *Proc. Chem.* 18 162–168. 10.1016/j.proche.2016.01.025

[B55] Tsou L. K. Lara-Tejero M. Rosefigura J. Zhang Z. J. Wang Y. C. Yount J. S. (2016). Antibacterial flavonoids from medicinal plants covalently inactivate type iii protein secretion substrates. *J. Am. Chem. Soc.* 138 2209–2218. 10.1021/jacs.5b11575 26847396 PMC4831573

[B56] Van Bambeke F. Michot J. M. Van Eldere J. Tulkens P. M. (2005). Quinolones in 2005: An update. *Clin. Microbiol. Infect.* 11 256–280. 10.1111/j.1469-0691.2005.01131.x 15760423

[B57] Varma J. K. Greene K. D. Ovitt J. Barrett T. J. Medalla F. Angulo F. J. (2005). Hospitalization and antimicrobial resistance in *Salmonella* outbreaks, 1984-2002. *Emerg. Infect. Dis.* 11 943–946. 10.3201/eid1106.041231 15963293 PMC3367611

[B58] Velge P. Wiedemann A. Rosselin M. Abed N. Boumart Z. Chaussé A. M. (2012). Multiplicity of *Salmonella* entry mechanisms, a new paradigm for *Salmonella* pathogenesis. *Microbiologyopen* 1 243–258. 23170225 10.1002/mbo3.28 PMC3496970

[B59] Vergalli J. Atzori A. Pajovic J. Dumont E. Malloci G. Masi M. (2020). The challenge of intracellular antibiotic accumulation, a function of fluoroquinolone influx versus bacterial efflux. *Commun. Biol.* 3:198. 10.1038/s42003-020-0929-x PMC7189378 32346058

[B60] Vergalli J. Dumont E. Cinquin B. Maigre L. Pajovic J. Bacqué E. (2017). Fluoroquinolone structure and translocation flux across bacterial membrane. *Sci. Rep.* 7:9821. 10.1038/s41598-017-08775-4 PMC5575017 28851902

[B61] Wang S. Li Z. Yu Y. Xu J. (2017a). Folding membrane proteins by deep transfer learning. *Cell Syst.* 5 202–211.e3. 28957654 10.1016/j.cels.2017.09.001 PMC5637520

[B62] Wang S. Sun S. Li Z. Zhang R. Xu J. (2017b). Accurate de novo prediction of protein contact map by ultra-deep learning model. *PLoS Comput. Biol.* 13:e1005324. 10.1371/journal.pcbi.1005324 28056090 PMC5249242

[B63] Wang S. Sun S. Xu J. (2018). Analysis of deep learning methods for blind protein contact prediction in CASP12. *Prot. Struct. Funct. Bioinforma.* 86(Suppl. 1) 67–77. 10.1002/prot.25377 28845538 PMC5871922

[B64] Worthington R. J. Melander C. (2013). Combination approaches to combat multidrug-resistant bacteria. *Trends Biotechnol.* 31 177–184. 10.1016/j.tibtech.2012.12.006 23333434 PMC3594660

[B65] Wu J. Pugh R. Laughlin R. C. Andrews-Polymenis H. McClelland M. Bäumler A. J. (2014). High-throughput assay to phenotype *salmonella enterica typhimurium* association, invasion, and replication in macrophages. *J. Vis. Exp.* 14:51759. 10.3791/51759 PMC4500590 25146526

[B66] Xiao Z. P. Ma T. W. Fu W. C. Peng X. C. Zhang A. H. Zhu H. L. (2010). The synthesis, structure and activity evaluation of pyrogallol and catechol derivatives as *Helicobacter* pylori urease inhibitors. *Eur. J. Med. Chem.* 45 5064–5070. 10.1016/j.ejmech.2010.08.015 20801557

[B67] Xu J. (2019). Distance-based protein folding powered by deep learning. *Proc. Natl. Acad. Sci. U.S.A.* 116 16856–16865. 10.1073/pnas.1821309116 31399549 PMC6708335

[B68] Xu J. Wang S. (2019). Analysis of distance−based protein structure prediction by deep learning in CASP13. *Prot. Struct. Funct. Bioinform.* 87 1069–1081. 10.1002/prot.25810 31471916

[B69] Yamasaki S. Nagasawa S. Hayashi-Nishino M. Yamaguchi A. Nishino K. (2011). AcrA dependency of the AcrD efflux pump in *Salmonella enterica* serovar *typhimurium*. *J. Antibiot (Tokyo).* 64 433–437. 10.1038/ja.2011.28 21505470

[B70] Yanagawa Y. Yamamoto Y. Hara Y. Shimamura T. (2003). A combination effect of epigallocatechin gallate, a major compound of green tea catechins, with antibiotics on *Helicobacter* pylori growth in vitro. *Curr. Microbiol.* 47 244–249. 10.1007/s00284-002-3956-6 14570277

[B71] Yang J. M. Chen C. C. (2004). Gemdock: a generic evolutionary method for molecular docking. *Prot. Struct. Funct. Genet.* 55 288–304. 10.1002/prot.20035 15048822

